# TransDFL: Identification of Disordered Flexible Linkers in Proteins by Transfer Learning

**DOI:** 10.1016/j.gpb.2022.10.004

**Published:** 2022-10-19

**Authors:** Yihe Pang, Bin Liu

**Affiliations:** 1 School of Computer Science and Technology, Beijing Institute of Technology, Beijing 100081, China; 2 Advanced Research Institute of Multidisciplinary Science, Beijing Institute of Technology, Beijing 100081, China

**Keywords:** Intrinsically disordered protein, Disordered flexible linker, False positive rate, Computational predictor, Transfer learning

## Abstract

**Disordered flexible linkers** (DFLs) are the functional disordered regions in proteins, which are the sub-regions of intrinsically disordered regions (IDRs) and play important roles in connecting domains and maintaining inter-domain interactions. Trained with the limited available DFLs, the existing DFL predictors based on the machine learning techniques tend to predict the ordered residues as DFLs, leading to a high **falsepositive rate** (FPR) and low prediction accuracy. Previous studies have shown that DFLs are extremely flexible disordered regions, which are usually predicted as disordered residues with high confidence [*P*(*D*) > 0.9] by an IDR predictor. Therefore, transferring an IDR predictor to an accurate DFL predictor is of great significance for understanding the functions of IDRs. In this study, we proposed a new predictor called TransDFL for identifying DFLs by transferring the RFPR-IDP predictor for IDR identification to the DFL prediction. The RFPR-IDP was pre-trained with IDR sequences to learn the general features between IDRs and DFLs, which is helpful to reduce the false positives in the ordered regions. RFPR-IDP was fine-tuned with the DFL sequences to capture the specific features of DFLs so as to be transferred into the TransDFL. Experimental results of two application scenarios (prediction of DFLs only in IDRs or prediction of DFLs in entire proteins) showed that TransDFL consistently outperformed other existing DFL predictors with higher accuracy. The corresponding web server of TransDFL can be freely accessed at http://bliulab.net/TransDFL/.

## Introduction

Intrinsically disordered regions (IDRs) are protein regions without stable three-dimensional (3D) structures, which are particularly common among eukaryotic organisms and viral proteomes [1]. Although the IDRs lack well-defined 3D structures, they carry out many critical functions, such as transcriptions, signal transmission, post-translational modifications, and multi-protein aggregation [2]. The functions of IDRs derive either from binding to molecular partners (such as DNA, RNA, and proteins) or directly from their native disordered states, where the former is called binding functions and the latter is called non-binding functions [3]. According to the DisProt database [4], about 75% of the non-binding functions are disordered flexible linkers (DFLs) [5], [6]. DFLs serve as the linkers in multidomain proteins characterized by extremely structural flexibility, and can be located between inter- and intra-domain, which are different from generic linkers [5], [7], [8], [9], [10]. DFLs play essential roles for intramolecular allosteric regulation [2], [11] and phase separation [12]. Identification of DFLs is crucial for comprehensively studying IDR functions. Experimental annotation of DFLs primarily relies on X-ray crystallography, nuclear magnetic resonance (NMR) spectroscopy, and circular dichroism. In order to efficiently identify the DFLs, two computational methods have been developed only based on the protein sequences, including DFLpred [5] and APOD [6]. DFLpred identifies the DFLs via combining the logistic regression (LR) and four sequence-based features, including structure domain propensities, putative disordered regions, and two properties of spiral and turn formation. APOD incorporates various sequence profile features into support vector machines (SVMs) to further improve the predictive performance, such as evolutionary conservation and relative solvent accessible area.

These predictors successfully incorporate various sequence profile features for DFL prediction. However, DFLs are continuous regions in proteins, sharing global sequence patterns along the whole protein [5]. The global features of DFLs should be incorporated into the DFL predictors. Furthermore, DFLs are the sub-regions of IDRs, while sequences with unannotated disordered regions are common in nature [13], [14], [15]. As a result, DFL predictors tend to predict the ordered residues as DFLs, resulting in high false positive rate (FPR) and low prediction accuracy.

**Figure 1:**
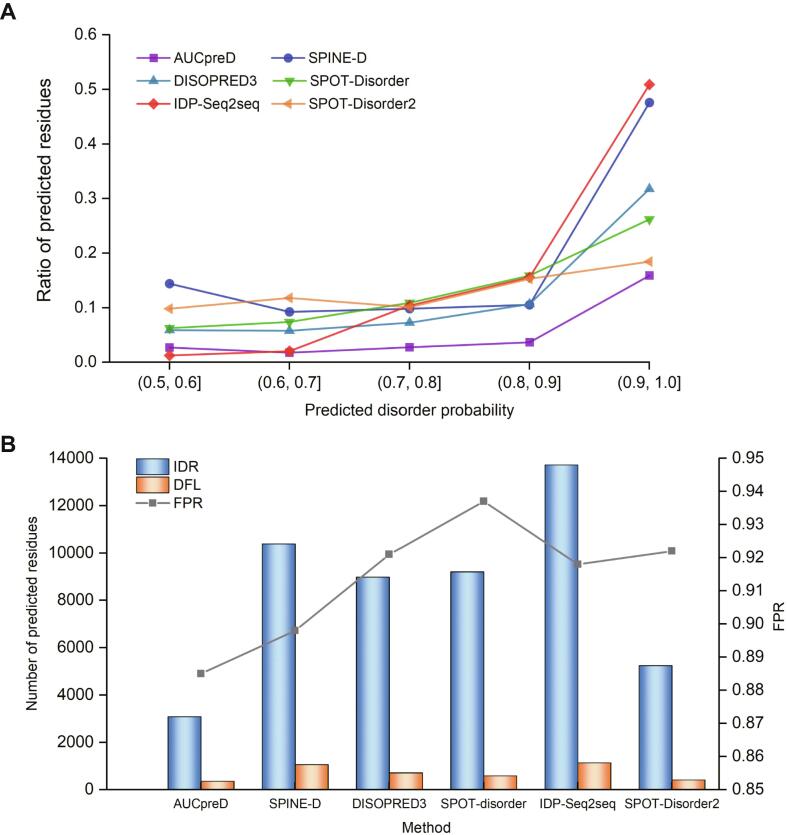
**Applying the IDR predictorsto DFLs** **A.** The relationships between the true DFLs and their probability scores predicted by IDR predictors. DFLs are preferred to be confidently predicted by IDR predictors with higher disordered probabilities, *i.e.*, extremely disordered state *P*(*D*) > 0.9. **B.** Histogram showing the number of predicted IDRs and DFLs by different IDR predictors with probabilities between 0.9 and 1.0. The line shows the corresponding FPR of each predictor, which equals the ratio of non-DFLs in predicted IDRs. DFL, disordered flexible linker; IDR, intrinsically disordered region; FPR, false positive rate.

According to the recent Critical Assessment of protein Intrinsic Disorder prediction (CAID) experiment reports [16], great efforts have been made by researchers for the development of IDR predictors. Figure 1 shows DFL prediction results on the DFL dataset TE82 [6] predicted by six state-of-the-art IDR predictors, including AUCpreD [17], SPINE-D [18], DISOPRED3 [19], SPOT-Disorder [20], IDP-Seq2Seq [15], and SPOT-Disorder2 [14]. We can see that DFLs can be predicted with high disordered probabilities [*i.e.*, extremely disordered state *P*(*D*) > 0.9] by different IDR predictors, providing an opportunity to predict the DFLs based on an IDR predictor (Figure 1A).

**Figure 2:**
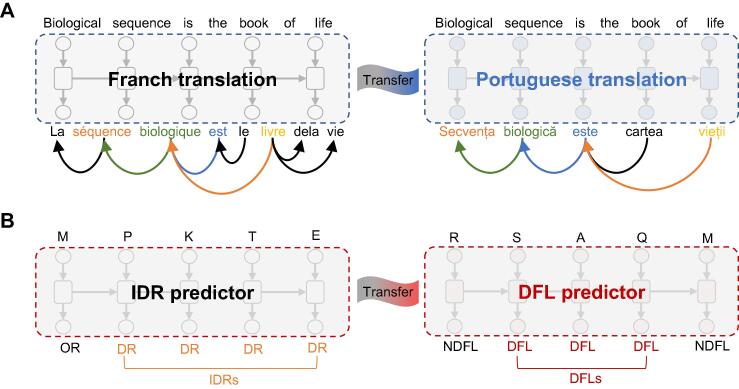
**Comparison between the transfer learning frameworks for machine translation and DFL prediction** **A.** Linguistic commonalities learned from the language translation pairs, *i.e.*, the English–French parallel corpus can be adapted to the English–Portuguese translation by transfer learning. **B.** In DFL prediction, the IDR predictor RFPR-IDP trained with the IDR datasets was transferred to predict the DFLs by transfer learning. OR, ordered region; DR, disordered region; NDFL, non-DFL.

The information of disordered regions and functions are both encoded in their primary sequences, similar to the source language and target language sharing the same semantic [21] in the field of machine translation. For example, French and Portuguese are both from the Romance language family sharing similar grammatical structures, and the pre-trained French translation model can be transferred to Portuguese translation via transfer learning [22], [23] (Figure 2A). Motivated by the similarities between protein sequences and natural languages, we treated the IDR prediction as the French translation and the DFL prediction as the Portuguese translation (Figure 2B) according to the discussion of predictive correlations between IDRs and DFLs in Figure 1. A new DFL predictor was proposed called TransDFL, which was transferred from an IDR predictor by the transfer learning technology. The IDR predictor RFPR-IDP [24] was pre-trained with the IDR data to learn the common features between DFLs and IDRs, and then it was transferred to DFL prediction by fine-tuning so as to capture the specific features of DFLs. The proposed TransDFL has the following advantages: (1) the predicted model employs the sequence labeling method by combining bi-directional long short-term memory (Bi-LSTM) neural network and convolutional neural network (CNN), which models the protein as a whole and captures the local and long-range interaction features among residues; (2) the disordered features learned from the pre-trained IDR predictor by transfer learning can reduce the incorrectly predicted DFL residues in the ordered regions, leading to a lower FPR.

We evaluated the performance of different DFL predictors in two scenarios: prediction of DFLs only in the IDRs (situation-I) and prediction of DFLs in the entire proteins (situation-II). Experimental results showed that TransDFL consistently outperformed existing predictors. Furthermore, the corresponding web server of TransDFL was established, which can be accessed at http://bliulab.net/TransDFL/.

## Method

### Datasets

In the pre-training phase, the IDR benchmark dataset was used to train the RFPR-IDP predictor [15]. To avoid the redundancy between the source and target domains, proteins sharing > 25% similarity with any protein in the DFL datasets (TR166, TE82, and TE64) were removed from the IDR benchmark dataset by using the BLASTClust search tool [25], leading to 2645 training IDR sequences and 1077 validation IDR sequences.

In fine-tuning phase, the TR166 [6] DFL benchmark dataset collected by Peng et al. [6] was used for model fine-tuning, and any two proteins in the dataset share sequence similarity < 25%. We randomly divided the DFL benchmark dataset into five subsets. Four of the subsets with 133 sequences were randomly selected as the training dataset for fine-tuning the model parameters, and the remaining subset with 33 sequences was employed as the validation dataset for model selection. This way ensures that there is no redundancy between the validation and training datasets.

In this study, TE82 and TE64 independent test sets were used for the performance evaluation of different DFL predictors. The TE82 test set has 82 sequences collected from the DisProt database (version 8.0) by Peng et al. [6], and the sequence similarity between the TE82 and TR166 datasets is < 25%. We constructed a new independent test set TE64 from the latest released DisProt database (version 9.0, September 2021) [4], [26]. Following the previous annotation protocols [5], [6], IDR proteins that have DFL functionally annotated regions in the database were collected as “DFL proteins”. To reduce data redundancy and avoid overestimating the predictive performance, only the sequences sharing < 25% similarity with any protein in TE82, TR166, and IDR benchmark datasets were included in TE64. Finally, 64 sequences were collected as the TE64 independent test set.

All benchmark datasets used in this study can be downloaded online at http://bliulab.net/TransDFL/benchmark/.

### The overview of TransDFL predictor

**Figure 3:**
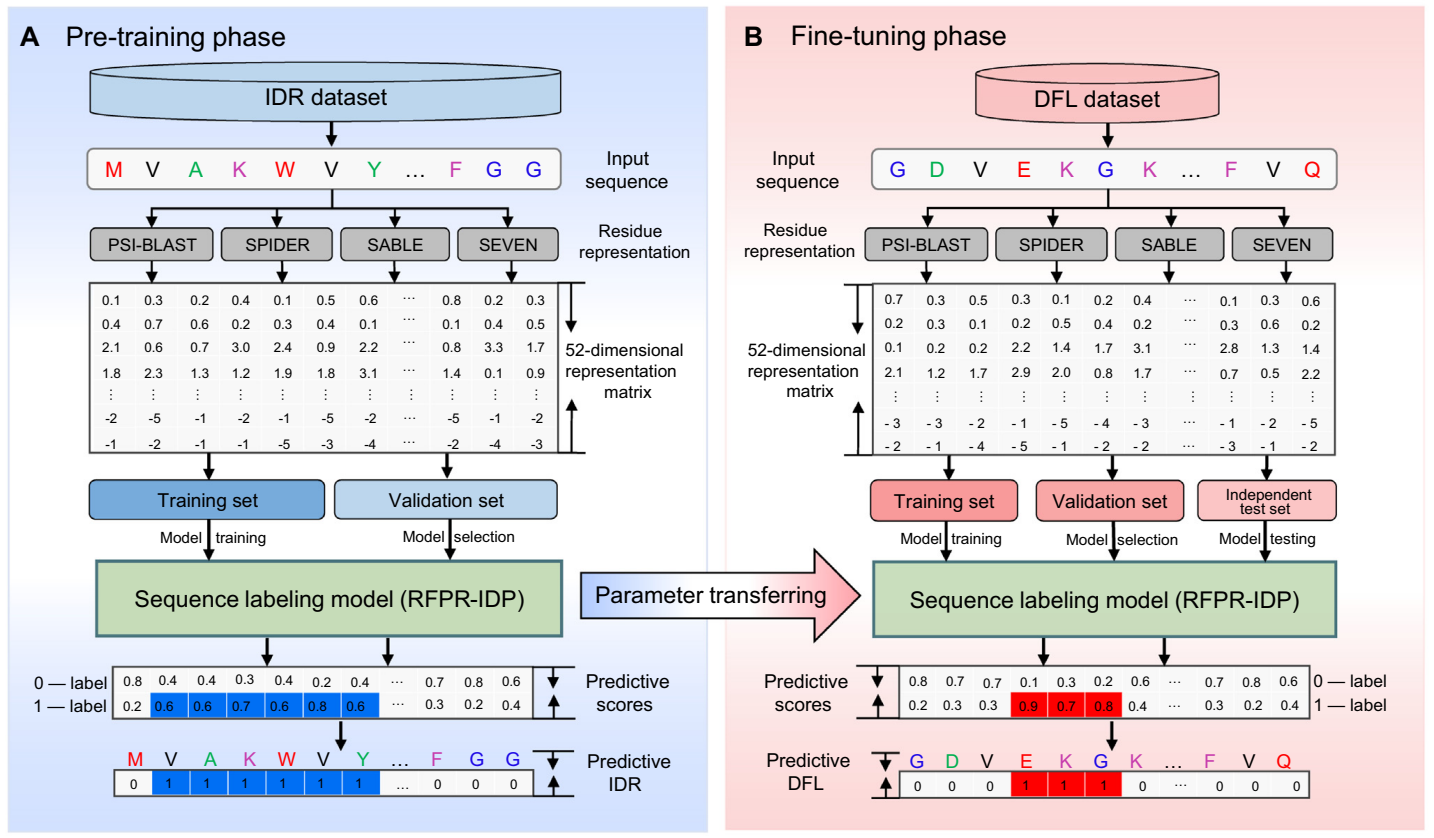
**The flowchart of the TransDFL predictor** **A.** In the pre-training phase, the IDR dataset was used for pre-training the sequence labeling model of the IDR predictor RFPR-IDP. **B.** In the fine-tuning phase, the DFL dataset was used to fine-tune the sequence labeling model for DFL prediction through transfer learning.

The overall flowchart of TransDFL is shown in Figure 3.

#### Sequence representation

In this study, the state-of-the-art IDR predictor RFPR-IDP [24] was employed to transfer into the DFL predictor. Two sequential features were used to represent the sequence in RFPR-IDP, including seven commonly used physicochemical properties [27] (steric parameter, polarizability, volume, hydrophobicity, isoelectric point, helix probability, and sheet probability), and position specific score matrix (PSSM) features generated by the PSI-BLAST tool [25] searching against the nrdb90 database (downloaded from https://ftp.ebi.ac.uk/pub/databases/nrdb90/). In this study, we also incorporated two additional features into RFPR-IDP so as to more comprehensively represent the DFL sequences, including the secondary structure (SS) features generated by the SPIDER tool [28], [29], and the solvent accessibility (SA) features generated by the SABLE tool [30], [31]. The linear combination of the 4-dimensional SS features, 1-dimensional SA feature, 7-dimensional physicochemical features (SEVEN), and 40-dimensional PSSM features, leads to a feature vector with 52 dimensions for representing a residue $R_{i}$ as:

(1) $$F_{i}={\left[f_{1},f_{2},\cdots ,f_{52}\right]}^{T}$$

The four features provide complementary information, and their combination leads to the best prediction performance (Table S1). Following previous studies [5], [6], the local sliding window was applied to represent the residues.

#### The sequence labeling model transferred to TransDFL

**Figure 4:**
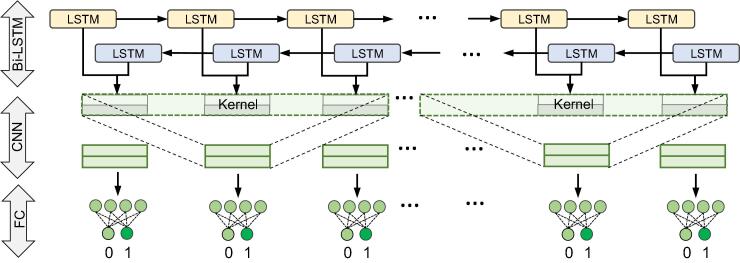
**The sequence labeling model architecture transferred to TransDFL** FC, fully-connected; CNN, convolutional neural network; LSTM, long short-term memory; Bi-LSTM, bi-directional long short-term memory.

The sequence labeling model is able to incorporate the correlation among adjacent residues and capture the interaction features of residues along the whole proteins. Two IDR predictors IDP-Seq2Seq [15] and RFPR-IDP [24] based on the sequence labeling model were used to be transferred to TransDFL. However, due to the insufficient number of DFL training sequences, the IDP-Seq2Seq using a more complex network structure is not suitable. Therefore, the RFPR-ID predictor by a combination of Bi-LSTM and CNN is more suitable for transferring to TransDFL. The model architecture is shown in Figure 4. The Bi-LSTM layer with a forward and a backward LSTM network layer was adopted to capture the global correlation features. For each residue $R_{i}$, the correlation feature vector $H_{i}$ is calculated by [24]:

(2) $$H_{i}=\left[\vec{h_{i}\;};\;\overset{↼}{h_{i}\;}\right]$$

(3) $$\overset{↼}{h_{i}},\overset{↼}{c_{i}}=LSTM_{f}(win_{k}F_{i},\vec{h_{i-1}};\vec{c_{i-1}})$$

(4) $$\overset{↼}{h_{i}},\;\overset{↼}{c_{i}}={\mathrm{LSTM}}_{b}\left({\mathrm{win}}_{k}F_{i},\overset{↼}{h_{i-1}};\;\overset{↼}{c_{i-1}}\right)$$

where $\vec{h_{i}}$ and $\overset{\leftarrow }{h_{i}}$ represent the forward and backward output feature vectors of $R_{i}$, respectively. The ${\mathrm{win}}_{k}F_{i}$ is the feature representation vector of $R_{i}$, which is the combination of the corresponding feature vectors of target residue $R_{i}$ and its *k* − 1 neighboring residues.

The convolutional layer was used to capture the local correlation features $H_{i}^{'}$ of $R_{i}$:

(5) $$H_{i}^{'}=conv\left(W,H_{\text{i}}\right)+b$$

where W is the convolutional kernel and b is the bias parameter matrix.

Then, a fully-connected layer was used to predict the label of each residue, mapping the output feature vector $H_{i}^{'}$ from the CNN layer to a probability score $p_{i}$ of $R_{i}$ being a positive residue, which is calculated by [32]:

(6) $$O_{i}^{1}=tanh\left(W_{1}^{1}H_{i}^{'}\;+b_{1}^{1}\right)$$

(7) $$O_{i}^{2}=tanh\left(W_{1}^{2}O_{i}^{1}+b_{1}^{2}\right)$$

(8) $$p_{i}=softmax\left(W_{2}O_{i}^{2}+b_{2}\right)$$

where $O_{i}^{1}$ and $O_{i}^{2}$ represent the output vectors of the first and second fully-connected layers, respectively; $W_{1}^{1}$, $W_{1}^{2}$, and $W_{2}$ are the trainable weight parameter vectors; $b_{1}^{1}$, $b_{1}^{2}$, and $b_{2}$ are the trainable bias parameter vectors; *tanh* is the hyperbolic tangent activation function [33]; and *softmax* is the soft argmax activation function [34].

#### The pre-training phase

In the pre-training phase, the RFPR-IDP predictor was pre-trained with the IDR dataset (Figure 3A). The pre-trained model was optimized based on the binary cross entropy loss function calculated by [35]:

(9) $$loss=-\sum_{i=1}^{L}\left(y_{i}log\left(p_{i}\right)+{(1-y}_{i}\right)log\left(1-p_{i}\right))$$

where *L* is the length of a sequence, $p_{i}$ is the predictive probability score of residue $R_{i}$ being an IDR residue [Equation (8)], and $y_{i}$ is the corresponding real label (0 or 1). All the model parameters were optimized by minimizing the loss function value on the IDR validation set. The model pre-trained with the source domain IDR dataset learns the common characteristics shared with IDRs and DFLs, which can be used for DFL prediction by transfer learning. The hyperparameters of RFPR-IDP in the pre-training phase are given in Table S3.

#### The fine-tuning phase

Because DFLs are the extremely flexible disordered regions predicted by IDR predictors with high probabilities, this pattern can be transferred to identify the DFLs via transfer learning (Figure 3B). Different from the model directly trained with the target dataset with a limited number of samples, the model fine-tuned based on the pre-trained parameters avoids over-fitting, and improves the prediction performance in the target domain [36].

The weighted binary cross entropy loss function was employed in the fine-tuning phase:

(10) $$loss=-\sum_{i=1}^{L}\left[w\times y_{i}log\left(p_{i}\right)+\left(1-w\right){(1-y}_{i}\right)log\left(1-p_{i}\right)]$$

where the *w* is the weight coefficient of DFL residue, optimized according to the best area under the receiver operating characteristic curve (AUROC) on the DFL validation dataset (Table S2). The model was implemented by the Tensorflow framework [37]. Adam algorithm [38] with a learning rate of 0.0008 was used for parameter optimization. All the parameters of the pre-trained RFPR-IDP model were fine-tuned on the validation set of the DFL benchmark dataset according to the minimum loss. The hyperparameters in the fine-tuning phase are given in Table S4.

#### Performance evaluation strategy

In this study, the AUROC was used to evaluate the overall performance of different methods [39], [40], [41]. Besides, following previous studies [6], [42], the Matthews correlation coefficient (MCC) [29], [43], precision (Pre), and recall (Rec) [44] were used to evaluate the predictive quality of a predictor:

(11) $$\left\{\begin{array}{c} \mathrm{MCC}=\frac{\mathrm{TP}\times TN-FP\times FN}{\sqrt{\left(TP+FP)(TP+FN)(TN+FP)(TN+FN\right)}}\\ Pre=\frac{\mathrm{TP}}{\mathrm{TP}+FP}\\ Rec=\frac{\mathrm{TP}}{\mathrm{TP}+FN} \end{array}\right)$$

where true positive (*TP*) is the number of DFL residues correctly predicted as DFLs, false positive (*FP*) is the number of non-DFL residues incorrectly predicted as DFLs, true negative (*TN*) is the number of non-DFL residues correctly predicted as non-DFLs, false negative (*FN*) is the number of DFL residues incorrectly predicted as non-DFLs. Given a threshold *thd*, a residue $R_{i}$ is classified as a DFL residue, if its predictive probability score $p_{i}\geq thd$. Otherwise, it is predicted as a non-DFL residue.

## Results and discussion

### Predicting the DFL residues in disordered regions

We compared the performance of TransDFL to the other two DFL predictors (DFLpred [5] and APOD [6]) on two independent test sets (TE82 and TE64). Following previous studies [5], [6], disordered regions without functional annotations and ordered regions were not evaluated (situation-I). The results of different predictors on the TE82 and TE64 datasets are shown in Tables 1 and 2, respectively. From these results, we can see that TransDFL outperforms DFLpred by 0.198 in terms of AUROC on the TE82 dataset, and achieves highly comparable performance with APOD. Particularly, the precision-recall (PR) curves (Figure S2) showed that the prediction results of TransDFL and APOD were complementary and their differences were significant (*P* = 0.001; Tables 1 and 2).

**Table 1:** **Performance comparison of TransDFL and otherDFLpredictors on the TE82 independent testset evaluated in situation-I**

**Method**	**Pre**	**Rec**	**MCC**	**AUROC**	***P* value**
DFLpred	0.337	0.179	0.145	0.637	1.360E−147
APOD	0.512	0.512	0.418	0.816	0.001
TransDFL	0.586	0.452	0.435	0.835	/

*Note*: The results of APOD and DFLpred were obtained from a previous study [6] evaluated on the same TE82 dataset. The *thd* of TransDFL was set as 0.16, which is equal to the ratio of DFL residues in the dataset. The *P* value was calculated by *t*-test based on the probabilities predicted by different methods. Pre, precision; Rec, recall; MCC, Matthews correlation coefficient; AUROC, area under receiver operating characteristic curve; DFL, disordered flexible linker.

**Table 2:** **Performance comparison of TransDFL and otherDFLpredictors on the TE64 independent testset evaluated in situation-I**

**Method**	**Pre**	**Rec**	**MCC**	**AUROC**	***P* value**
DFLpred	0.189	0.193	0.108	0.675	5.330E−15
APOD	0.195	0.600	0.223	0.751	0.001
TransDFL	0.207	0.722	0.273	0.784	/

*Note*: The results of APOD and DFLpred were calculated according to the predicted results obtained by running corresponding web servers. The *thd* of TransDFL was set as same as in Table 1. The *P* value was calculated by *t*-test based on the probabilities predicted by different methods.

### Predicting the DFL residues in the entire proteins

**Figure 5:**
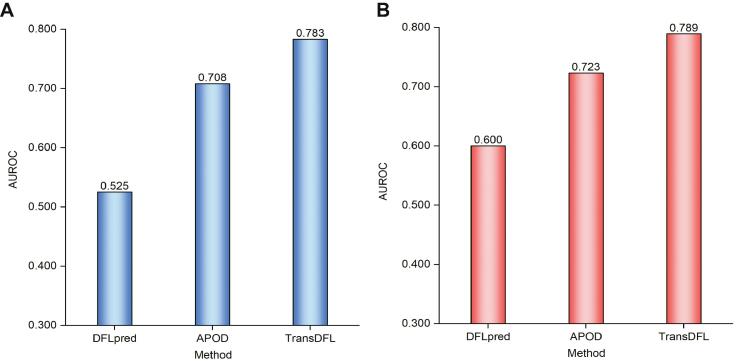
**Comparison of differentDFLpredictors in situation-II** **A.** AUROC values on the TE82 independent test set. **B.** AUROC values on the TE64 independent test set. AUROC, area under the receiver operating characteristic curve.

DFLs are flexible linkers in disordered regions. The existing two predictors (APOD and DFLpred) focus on predicting DFLs only in the functionally annotated disordered regions. However, the information on the disordered regions is not always available [15], [45], [46], [47]. In order to more comprehensively and fairly evaluate the performance of different methods, they were evaluated by identifying DFLs in the entire protein sequences in TE82 and TE64 datasets (situation-II). Their AUROC values are shown in Figure 5, and the PR curves and the area under the PR curve (AUPRC) are shown in Figure S3, from which we can see the followings: (1) compared with the results in situation-I, the performance of these three predictors decreased, indicating that predicting DFLs in the entire sequence is more challenging; (2) TransDFL obviously outperforms DFLpred and APOD on both two datasets in terms of AUROC.

### Performance comparison between TransDFL and IDR predictors

In order to investigate the performance of IDR predictors for predicting DFLs, we employed six state-of-the-art IDR predictors to identify DFLs. Because the average length of DFLs is 47 amino acids (aas) in the TR166 dataset (Figure S1), a target residue is considered as a DFL residue if its 46 neighboring residues are disordered residues (the target is in the middle). The results of different methods evaluated in two situations on the TE82 independent test set are shown in Tables S5 and S6. From these results, we can see that the six IDR predictors are not effective enough for identifying DFLs compared with the specific DFL predictor TransDFL in both two evaluation situations.

### Transfer learning obviously reduces the false positives

**Figure 6:**
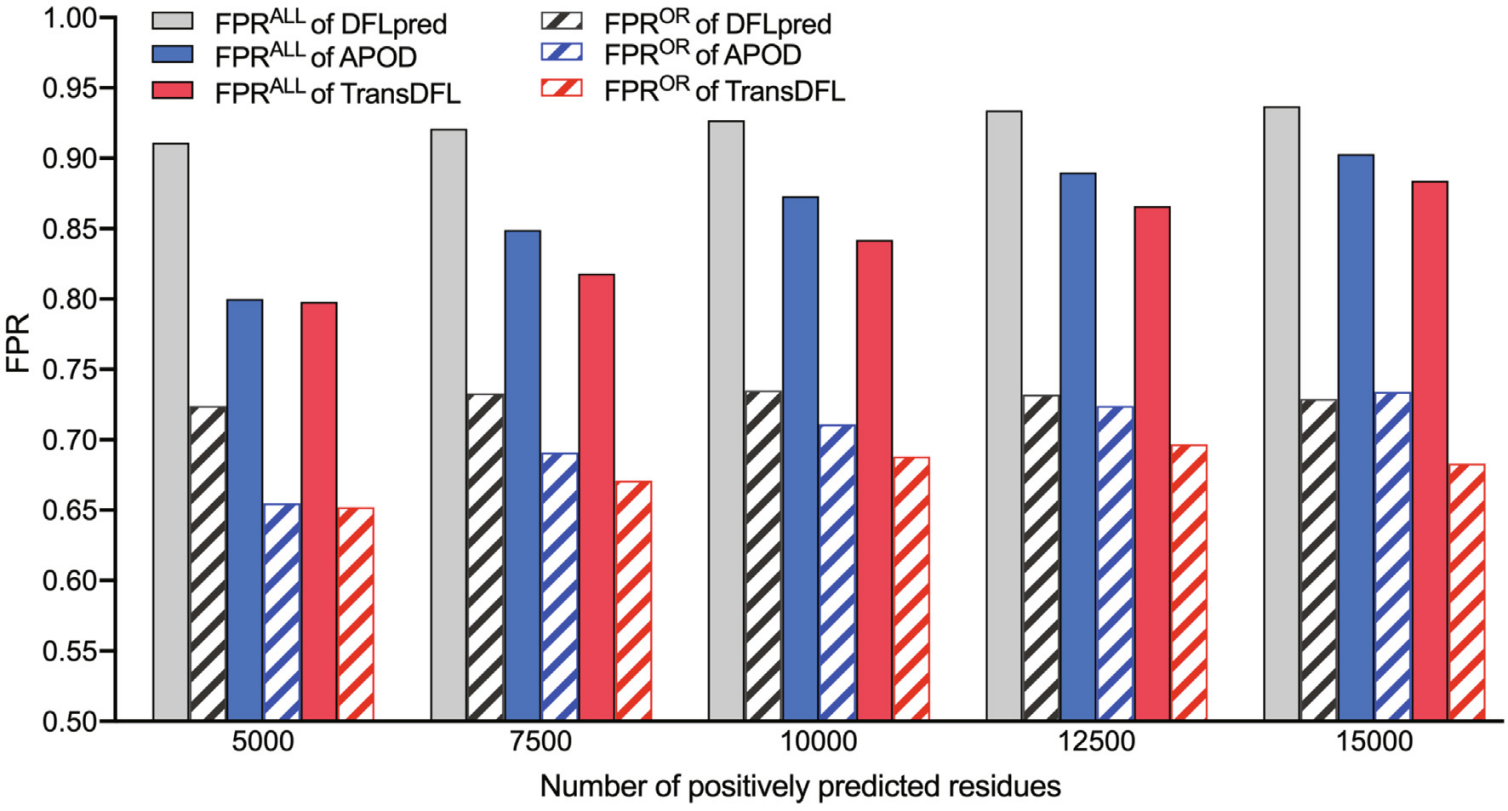
**Comparison ofFPRs of different predictors on the TE82 testset** FPR^ALL^, false positive rate in all predictions; FPR^OR^, false positive rate in the ordered region.

In order to explore the contribution of transfer learning to the performance improvement of TransDFL, we compared the FPR in all predictions (FPR^ALL^) and the FPR in the ordered region (FPR^OR^) of three different predictors. The FPR^OR^ is calculated as the ratio of the number of falsely predicted DFL residues in ordered regions to the number of all the positively predicted DFL residues, where the ordered residues are annotated according to the DisProt database (version 8.2). For fair evaluation, the FPRs of different predictors were compared under the same number of positively predicted residues. As shown in Figure 6, TransDFL achieves the lowest FPR^ALL^ and FPR^OR^. These results are not surprising because TransDFL employs the transfer learning framework pre-trained with the IDR dataset to capture the common characteristics between IDRs and DFLs. Therefore, compared with the other predictors only trained with DFLs and disordered residues, TransDFL can obviously reduce the number of incorrectly predicted DFL residues in the ordered regions so as to reduce the overall false positive predictions.

**Figure 7:**
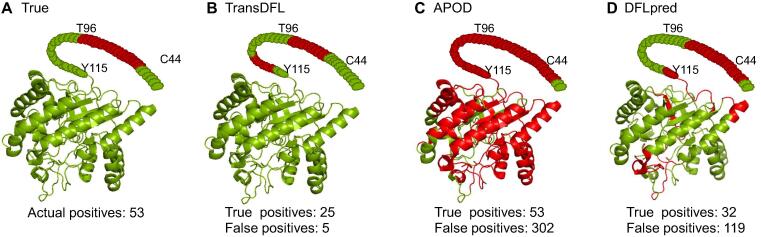
**Visualizationof predictive results** The predictive results of a protein (DisProt ID: DP01080; PDB ID: 1OCB) were visualized by the PyMOL software (https://pymol.org/2/). The true and predicted DFL residues are shown in red. **A.** The true DFLs. **B.** DFLs predicted by TransDFL. **C.** DFLs predicted by APOD. **D.** DFLs predicted by DFLpred.

The prediction results of a protein (DisProt ID: DP01080; PDB ID: 1OCB) from the TE82 independent test set obtained by different predictors were visualized by the PyMOL software (https://pymol.org/2/). As shown in Figure 7, although most of the DFLs can be correctly predicted by TransDFL, DFLpred, and APOD, the false positives predicted by TransDFL are obviously fewer than those predicted by DFLpred and APOD evaluated in situation-II. The false positives predicted by TransDFL are in the disordered regions near the true DFLs, while most of the false positives predicted by APOD and DFLpred are located in the ordered regions far away from the true DFLs. These results are fully consistent with the observations in Figure 6.

In order to explore the contribution of the model pre-trained with disordered proteins, we compared the predictive performance between the TransDFL model directly trained with DFLs (TransDFL-DT) and the fine-tuned model based on pre-training with IDRs (TransDFL). The evaluation results showed that TransDFL consistently outperformed TransDFL-DT on two independent test sets in both two situations (Table 3), indicating that transfer learning contributes to the predictive performance improvement of TransDFL.

**Table 3:** **Performance of TransDFL predictors based on different models**

**Dataset**	**Model**	**Situation-I**				**Situation-II**			
		**Pre**	**Rec**	**MCC**	**AUROC**	**Pre**	**Rec**	**MCC**	**AUROC**
TE82	TransDFL-DT	0.552	0.250	0.298	0.746	0.010	0.581	0.120	0.705
	TransDFL	0.207	0.722	0.273	0.789	0.149	0.727	0.241	0.783
TE64	TransDFL-DT	0.254	0.481	0.166	0.697	0.080	0.680	0.113	0.642
	TransDFL	0.275	0.518	0.289	0.784	0.207	0.722	0.273	0.789

*Note*: TransDFL-DT refers to the model directly trained with the DFL training set. TransDFL refers to the transferred model with pre-training on the IDR dataset. IDR, intrinsically disordered region.

### The sequence labeling model facilitates the stable performance on different lengths of DFL regions

**Figure 8:**
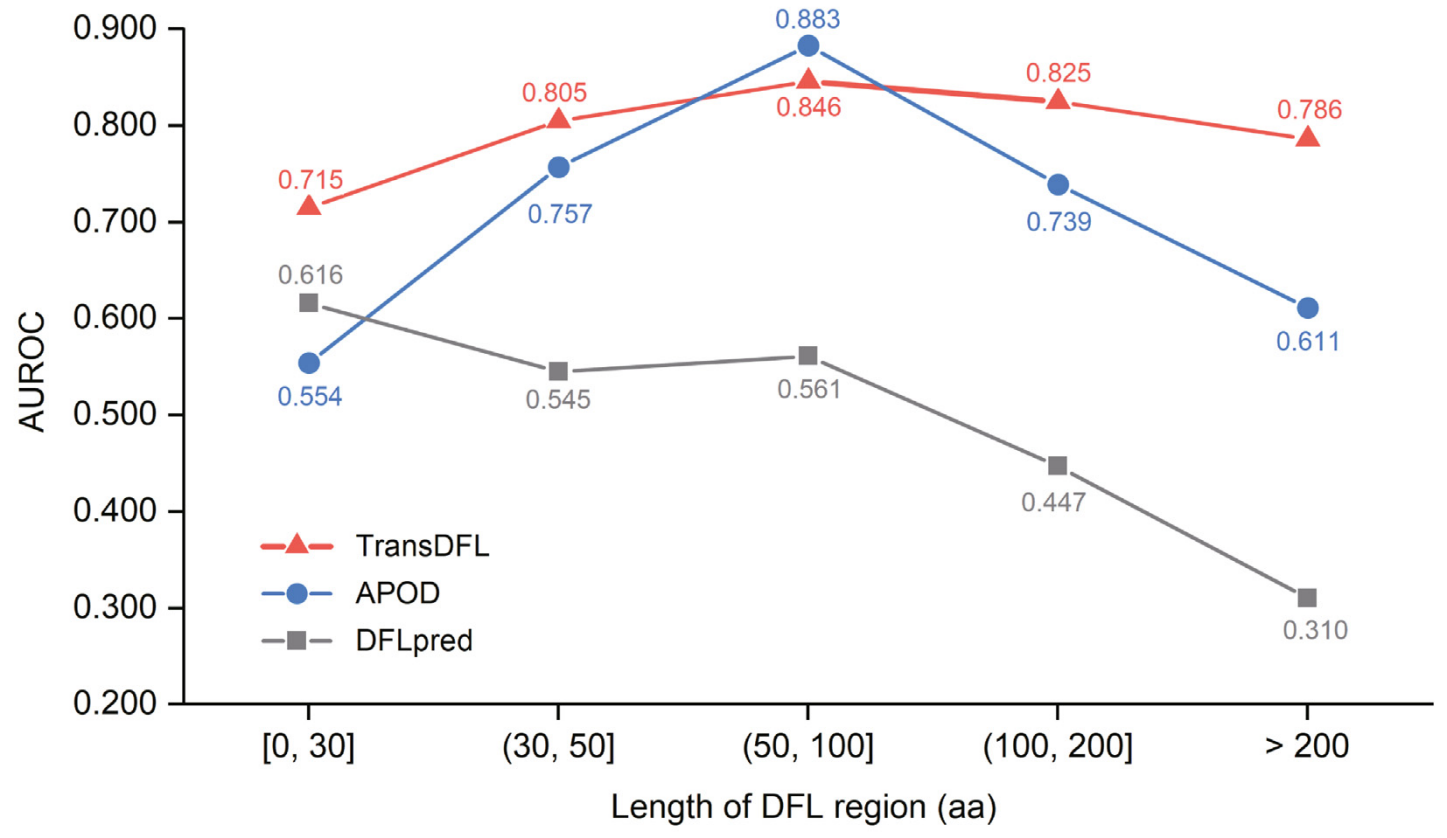
**Performancecomparison of TransDFL, APOD, and DFLpred for predicting proteins with different lengths of DFL regions** aa, amino acid.

In order to investigate the performance of TransDFL for predicting DFL regions with different lengths, we divided the protein sequences in the TE82 independent test set into five groups according to their DFL lengths. As shown in Figure 8, compared with APOD and DFLpred, TransDFL is insensitive to the lengths of DFL regions, and achieves better and more stable performance. There are two reasons. First, TransDFL employs the sequence labeling model based on deep learning technology, which is able to capture the local and global interactions among the residues and the sequence patterns of the DFLs. In contrast, all the other two classifiers are classification-based methods predicting each residue in a separate manner. Second, benefitting from the deep neural networks, the sequence labeling model in TransDFL captures the general disordered characteristics of DFLs from the large IDR dataset, which facilitates the DFL prediction.

### Lower FPR leads to better performance in the real-world application

**Figure 9:**
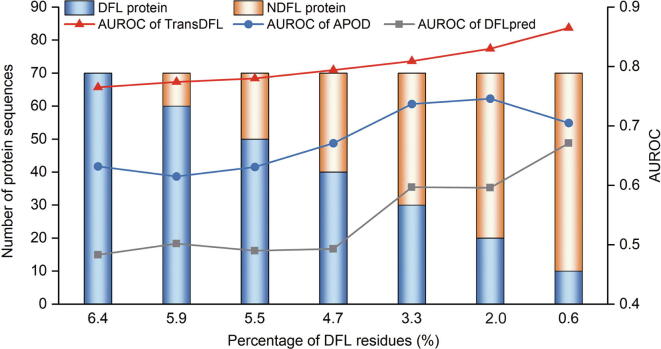
**Predictive results of TransDFL, APOD, and DFLpred on real-word datasets with different percentages of DFL residues**

According to the latest DisProt database, only 6.3% of the 4438 annotated disordered regions are DFLs [26]. There are even many more disordered proteins without DFLs in MobiDB [48]. As a result, the percentage of DFL residues is much lower than 6.3% in nature. Therefore, for real-world applications, it is important for a DFL predictor to deal with the extremely imbalanced problem (*i.e.*, the number of non-DFL residues is much higher than the number of DFL residues). In this regard, seven datasets were constructed based on TE82 and TE64 with different percentages of DFL residues. The performance of different predictors on the seven datasets is shown in Figure 9. We observed that TransDFL consistently outperformed both APOD and DFLpred, especially for the datasets with fewer DFL residues. These results indicate that TransDFL is able to solve the imbalanced problem, and therefore, it is more suitable for real-world applications.

## Conclusion

Inspired by the similarity between protein sequences and natural language sentences, we applied the transfer learning derived from the machine translation to the DFL identification, and a new predictor TransDFL was proposed. TransDFL was constructed by transferring the state-of-the-art IDR predictor RFPR-IDP into the current DFL predictor. It has the following advantages: (1) TransDFL employs the sequence labeling model to capture the global sequence patterns of DFLs; (2) benefitting from transfer learning, TransDFL is the first deep learning predictor for DFL prediction and achieves state-of-the-art performance with the lowest FPR. The web server of TransDFL was established, which can be freely accessed at http://bliulab.net/TransDFL.

## Code availability

The source code of TransDFL is available at https://ngdc.cncb.ac.cn/biocode/tools/BT007312.

## Data availability

The web server of TransDFL can be freely accessed at http://bliulab.net/TransDFL/.

## Competing interests

Both authors have declared no competing interests.

## CRediT authorship contribution statement

**Yihe Pang:** Methodology, Software, Validation, Formal analysis, Data curation, Writing – original draft, Writing – review & editing. **Bin Liu:** Conceptualization, Resources, Writing – review & editing, Supervision, Project administration, Funding acquisition. Both authors have read and approved the final manuscript.

## Supplementary material

Supplementary Figure S1 **The length distribution of DFLs on the TR166 dataset** Most DFL regions are relatively short. About 57% of DFL regions are shorter than 30 residues, and about 9% of DFL regions are longer than 100 residues. The average length of DFLs is 47. DFL, disordered flexible linkers.

Supplementary Figure S2 **Precision-recall curves of TransDFL and the other predictors in situation-I A.** TE82 dataset. **B.** TE64 dataset. AUPRC, the area under the Precision-Recall curve.

Supplementary Figure S3 **P****recision-recall curves of TransDFL and the other predictors in situation-II A.** TE82 dataset. **B.** TE64 dataset.

Supplementary Table S1 **The performance of TransDFL based on different feature combinations on DFL validation dataset**

Supplementary Table S2 **The performance of TransDFL based on different loss function weight coefficients on DFL validation dataset**

Supplementary Table S3 **The hyper-parameters of RFPR-IDP (pre-trained)**

Supplementary Table S4 **The hyper-parameters of TransDFL (fine-tuned)**

Supplementary Table S5 **The performance of 6 state-of-the-art IDR predictors for predicting DFLs on TE82 dataset (situation-I)**

Supplementary Table S6 **The performance of 6 state-of-the-art IDR predictors for predicting DFLs on TE82 dataset (situation-II)**
