# Supplementary figures and images for: TransDFL: Identification of Disordered Flexible Linkers in Proteins by Transfer Learning
Source: Genomics Proteomics Bioinformatics. 2022 Oct 19;21(2):359–69. doi: 10.1016/j.gpb.2022.10.004 (PMC10626177; doi:10.1016/j.gpb.2022.10.004)

## Slide 1
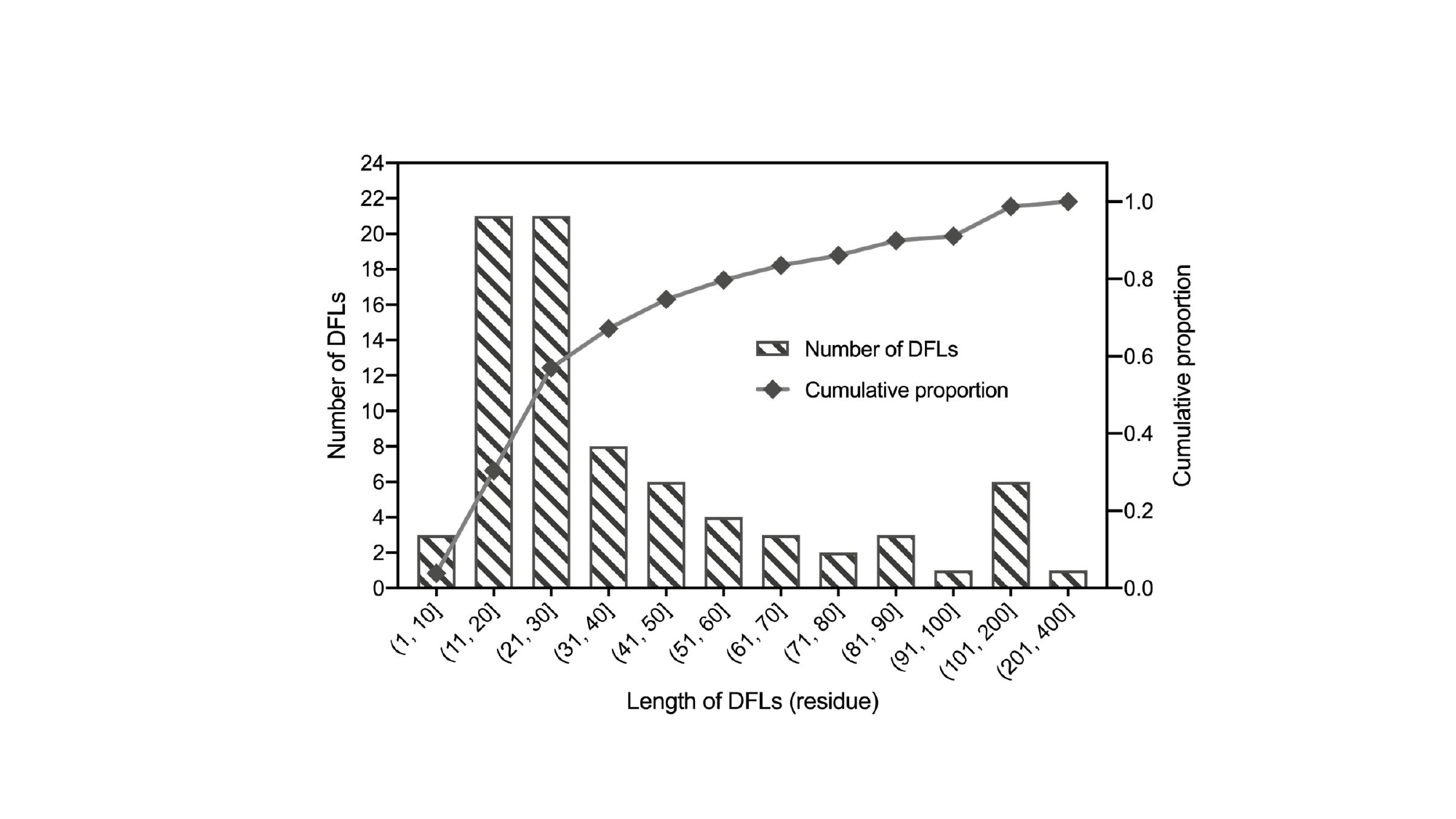

Supplement: Supplementary Figure S1 — The length distribution of DFLs on the TR166 dataset Most DFL regions are relatively short. About 57% of DFL regions are shorter than 30 residues, and about 9% of DFL regions are longer than 100 residues. The average length of DFLs is 47. DFL, disordered flexible linkers. [file mmc1.pptx]

A

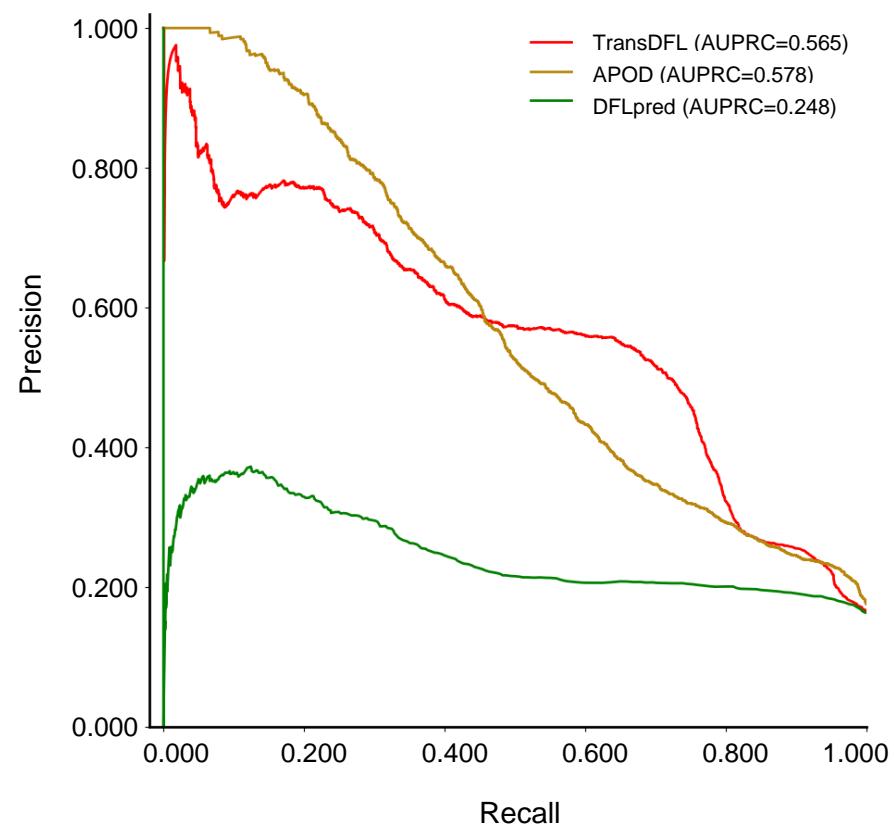

B

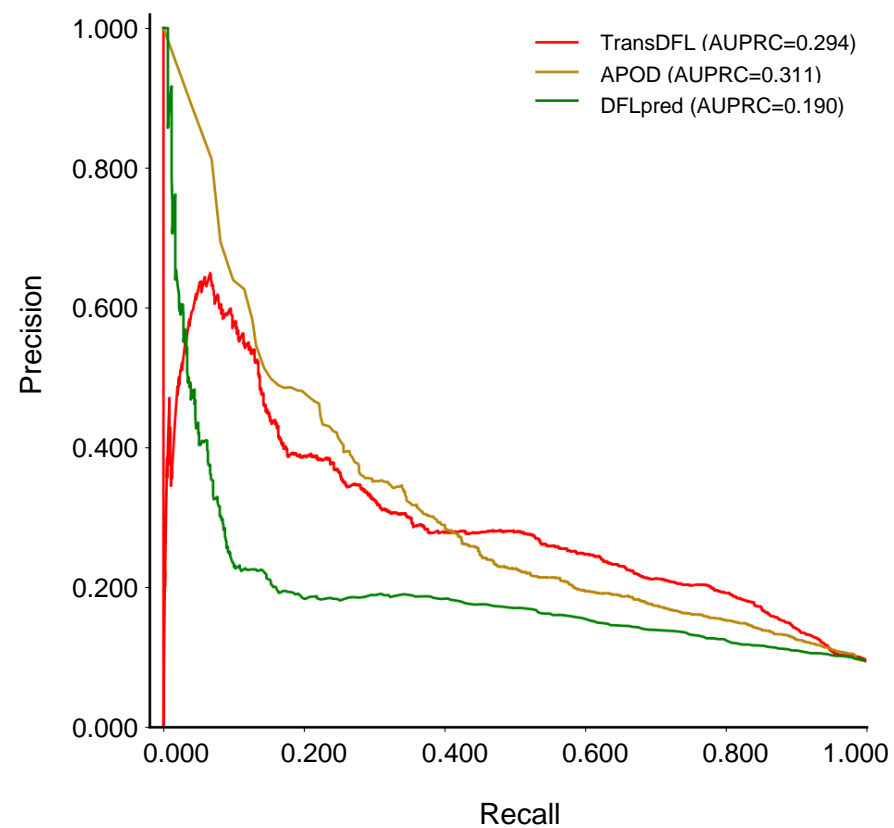

Supplement: Supplementary Figure S2 — Precision-recall curves of TransDFL and the other predictors in situation-I A. TE82 dataset. B. TE64 dataset. AUPRC, the area under the Precision-Recall curve. [file mmc2.pdf]

A

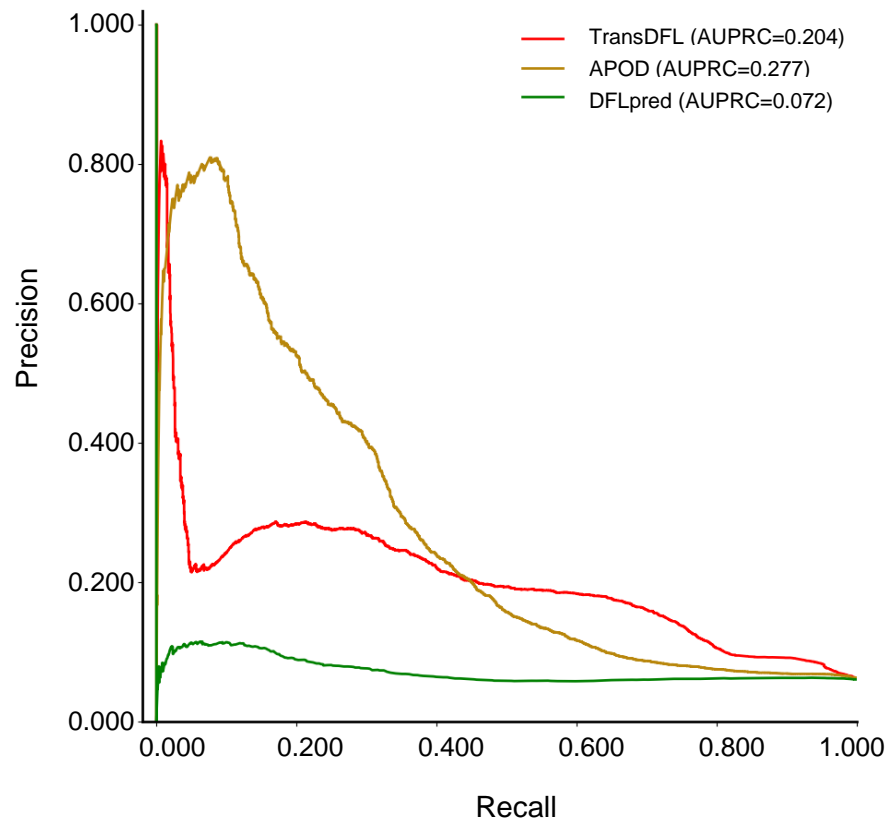

B

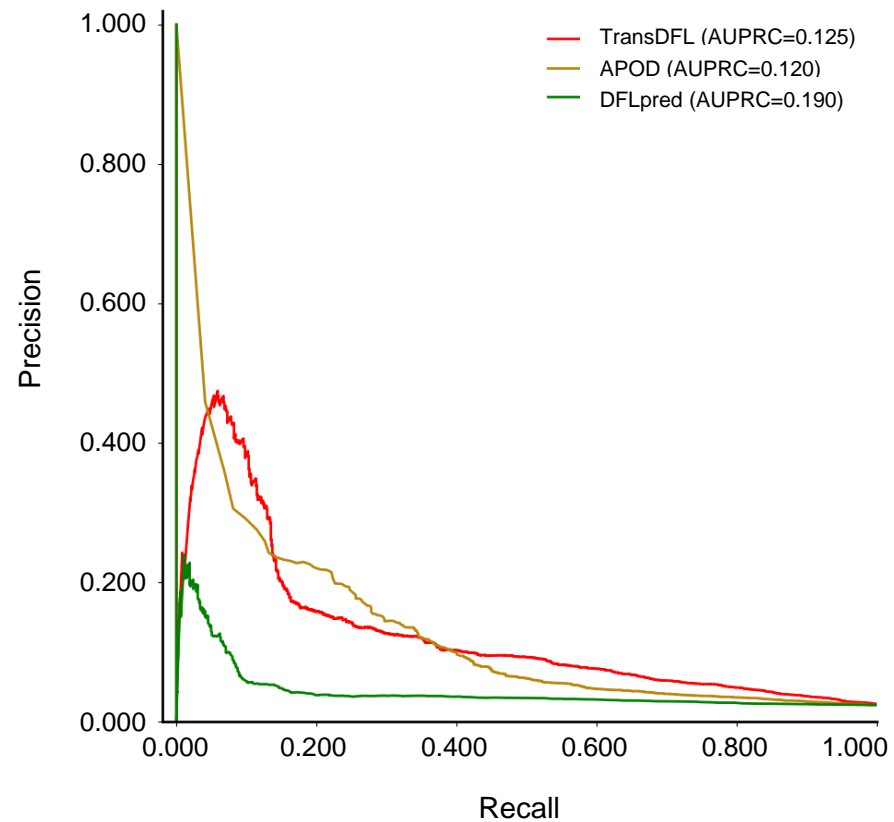

Supplement: Supplementary Figure S3 — Precision-recall curves of TransDFL and the other predictors in situation-II A. TE82 dataset. B. TE64 dataset. [file mmc3.pdf]
