# Supplementary material for: TransDFL: Identification of Disordered Flexible Linkers in Proteins by Transfer Learning
Source: Genomics Proteomics Bioinformatics. 2022 Oct 19;21(2):359–69. doi: 10.1016/j.gpb.2022.10.004 (PMC10626177; doi:10.1016/j.gpb.2022.10.004)
Supplement: Supplementary Table S1 — The performance of TransDFL based on different feature combinations on DFL validation dataset [file mmc4.docx]

**Table S1 The performance of TransDFL based on different combination of features on DFL validation dataset**

| **Features (dimension)** | **AUC** | |
| --- | --- | --- |
|  | **Situation-I** | **Situation-II** |
| PSSM (40) | 0.680 | 0.655 |
| Seven (7), SS (4), SA (1) | 0.778 | 0.689 |
| PSSM (40), Seven (7) | 0.752 | 0.702 |
| PSSM (40), Seven (7), SS (4) | 0.814 | 0.744 |
| PSSM (40), Seven (7), SS (4), SA (1) | 0.861 | 0.782 |

*Note*: AUC, area under receiver operating characteristic curve; DFL, disordered flexible linker; PSSM, position specific score matrix; Seven, 7-dimensional physicochemical features; SS, secondary structures; SA, solvent accessibility.
