# Supplementary material for: TransDFL: Identification of Disordered Flexible Linkers in Proteins by Transfer Learning
Source: Genomics Proteomics Bioinformatics. 2022 Oct 19;21(2):359–69. doi: 10.1016/j.gpb.2022.10.004 (PMC10626177; doi:10.1016/j.gpb.2022.10.004)
Supplement: Supplementary Table S2 — The performance of TransDFL based on different loss function weight coefficients on DFL validation dataset [file mmc5.docx]

**Table S2 The performance of TransDFL based on different loss function weight coefficients on DFL validation dataset**

| **Weight** | | **AUC** | |
| --- | --- | --- | --- |
| **Positive** | **Negative** | **Situation-I** | **Situation-II** |
| 0.5 | 0.5 | 0.833 | 0.761 |
| 0.6 | 0.4 | 0.861 | 0.782 |
| 0.7 | 0.3 | 0.826 | 0.755 |
| 0.8 | 0.2 | 0.805 | 0.718 |
