# Supplementary material for: TransDFL: Identification of Disordered Flexible Linkers in Proteins by Transfer Learning
Source: Genomics Proteomics Bioinformatics. 2022 Oct 19;21(2):359–69. doi: 10.1016/j.gpb.2022.10.004 (PMC10626177; doi:10.1016/j.gpb.2022.10.004)
Supplement: Supplementary Table S3 — The hyper-parameters of RFPR-IDP (pre-trained) [file mmc6.docx]

**Table S3 The hyper-parameters of RFPR-IDP (pre-trained)**

| **Hyper-parameter** | | **Details** |
| --- | --- | --- |
| Feature sliding window | | 9 |
| Batch_size | | 25 |
| Learning_rate | | 0.005 |
| Input feature dimension | | 52 |
| Bi-LSTM layer | Forward LSTM layer | RNN_cell: LSTM |
|  |  | Hidden size of the RNN cell: 148 |
|  | Backward LSTM layer | RNN_cell: LSTM |
|  |  | Hidden size of the RNN cell: 148 |
| CNN layer | Filter_size | [9,52,1,592] |
| Full connected layer | Hidden layer 1 | Units: 2 |
|  |  | Activation: Tanh |
| Output layer | | Activation: Softmax |

*Note*: CNN, convolutional neural network; Bi-LSTM, bi-directional long short-term memory neural network.
