# Supplementary material for: TransDFL: Identification of Disordered Flexible Linkers in Proteins by Transfer Learning
Source: Genomics Proteomics Bioinformatics. 2022 Oct 19;21(2):359–69. doi: 10.1016/j.gpb.2022.10.004 (PMC10626177; doi:10.1016/j.gpb.2022.10.004)
Supplement: Supplementary Table S5 — The performance of 6 state-of-the-art IDR predictors for predicting DFLs on TE82 dataset (situation-I) [file mmc8.docx]

**Table S5 The performance of 6 state-of-the-art IDR predictors for predicting DFLs on TE82 dataset (situation-I)**

| **Predictor** | **Pre** | **Rec** | **F1** |
| --- | --- | --- | --- |
| TransDFL | 0.586 | 0.452 | 0.510 |
| SPINE-D | 0.132 | 0.550 | 0.213 |
| IDP-Seq2seq | 0.114 | 0.468 | 0.183 |
| AUCpreD | 0.087 | 0.245 | 0.128 |
| SPOT-Disoreder | 0.098 | 0.349 | 0.153 |
| DISOPRED3 | 0.089 | 0.286 | 0.136 |
| SPOT-Disoreder2 | 0.092 | 0.345 | 0.145 |

*Note*: Ranked by F1 value in descending order. Pre, precision; Rec, recall; F1, harmonic mean score of precision and recall.
