# Supplementary material for: TransDFL: Identification of Disordered Flexible Linkers in Proteins by Transfer Learning
Source: Genomics Proteomics Bioinformatics. 2022 Oct 19;21(2):359–69. doi: 10.1016/j.gpb.2022.10.004 (PMC10626177; doi:10.1016/j.gpb.2022.10.004)
Supplement: Supplementary Table S6 — The performance of 6 state-of-the-art IDR predictors for predicting DFLs on TE82 dataset (situation-II) [file mmc9.docx]

**Table S6 The performance of 6 state-of-the-art IDR predictors for predicting DFLs on TE82 dataset (situation-II)**

| **Predictor** | **Pre** | **Rec** | **F1** |
| --- | --- | --- | --- |
| TransDFL | 0.149 | 0.727 | 0.247 |
| SPINE-D | 0.098 | 0.550 | 0.166 |
| IDP-Seq2seq | 0.087 | 0.468 | 0.147 |
| SPOT-Disoreder | 0.079 | 0.349 | 0.129 |
| DISOPRED3 | 0.078 | 0.286 | 0.123 |
| SPOT-Disoreder2 | 0.075 | 0.345 | 0.123 |
| AUCpreD | 0.075 | 0.245 | 0.115 |
